# Postoperative anion gap associates with short- and long-term mortality after cardiac surgery: A large-scale cohort study

**DOI:** 10.3389/fcvm.2022.1024484

**Published:** 2022-10-12

**Authors:** Jiajing Li, Yu Tian, Lingzhi Wang, Jiayue Chen, Xiaoshu Chen, Huansen Huang, Yihao Li

**Affiliations:** ^1^Department of Anesthesiology, The Second Affiliated Hospital, Guangzhou Medical University, Guangzhou, China; ^2^Department of Emergency and Critical Care Medicine, Shanghai Pudong Hospital, Fudan University Pudong Medical Center, Shanghai, China

**Keywords:** anion gap, cardiac surgery, mortality, MIMIC-III, postoperative outcome

## Abstract

**Objective:**

To investigate whether postoperative anion gap (AG) is associated with short- and long-term mortality in patients following cardiac surgery.

**Materials and methods:**

We conducted a retrospective cohort study of adults who underwent cardiac surgery from the Medical Information Mart for Intensive Care - III database. The generalized additive model (GAM), logistic regression, and Cox regression were performed to assess the correlations between AG levels and in-hospital, 90-day, and 4-year mortality. Linear regression was used to evaluate the associations between AG and length of stay (LOS).

**Results:**

Totally, 6,410 subjects were enrolled in this study and classified into tertiles based on the initial AG levels. The GAM indicated a positive association between initial AG and in-hospital mortality after adjusting for potential confounders. Multivariate logistic analysis revealed that the risk of in-hospital mortality was higher among patients in tertile 2 (OR 2.05, 95% CI 1.11–3.76, *P* = 0.021) and tertile 3 (OR 4.51, 95% CI 2.57–7.91, *P* < 0.001) compared with those in tertile 1. For 90-day and 4-year mortality, multivariate Cox regression found similar associations between AG tertiles and mortality. The LOS in ICU and hospital also increased as AG tertiles increased. The *E*-value indicated robustness to unmeasured confounders.

**Conclusion:**

This study found a positive association between postoperative AG levels and short- and long-term mortality among patients after cardiac surgery. This relationship warrants further research.

## Introduction

According to statistics, around 2 million cardiac surgeries are performed worldwide each year (1). Cardiac surgery has seen great improvements over the last several decades; however, cardiac operation remains to be a high-risk procedure with a mortality rate of 2–6% (1). Under cardiac surgery, patients have a greater risk for postsurgical complications, which contribute to a higher risk of mortality and to increased hospital costs. Although many prognostic indicators for mortality after cardiac surgery have been identified, most of them could not be widely utilized in clinical practice. Exploring a simple and readily available clinical indicator for evaluating prognosis is still significant for patients after cardiac surgery.

As a common index of acid-base balance, serum anion gap (AG) has been utilized clinically for many years (2). It has been observed that AG was associated with various disorders and disease prognoses, such as hypertension (3), insulin resistance (4), and cardiorespiratory fitness (5). Moreover, recent studies showed that AG provided important clues for diagnosis or prognosis of many cardiovascular diseases (6–9). Increased AG was also demonstrated to correlate with cardiovascular mortality in general mortality (10); even a small increase of AG was related to enhanced mortality (11). As a particular type of operation, the cardiac procedure had a strong influence on hemodynamics and the internal environment; such impacts alter AG levels and pose huge challenges to reducing relative complications as well as mortality. Patients with altered AG may have different outcomes following cardiac surgery, but the association between different postoperative AG levels and mortality was rarely discussed.

Therefore, we sought to explore the relationship between AG and risk-adjusted surgical outcomes after cardiac surgery. We hypothesized that postoperative AG (maximum, minimum, and initial) was related to short- and long-term outcomes of cardiac surgery patients.

## Methods

### Data source

All data for this study were obtained from a massive critical care database named Medical Information Mart for Intensive Care III (MIMIC-III version 1.4). MIMIC-III database is freely accessible and has been well described previously (12). Briefly, this database comprises clinical data of ICU patients from Beth Israel Deaconess Medical Center between 2001 and 2012. The Institutional Review Board of the Massachusetts Institute of Technology has approved the MIMIC-III database. After accomplishing the online course from the National Institutes of Health and passing the Protecting Human Research Participants examination, we were approved to extract data from MIMIC-III (Record ID 46484149). The informed consent was waived in this retrospective study.

### Patient selection

According to the International Classification of Diseases, Ninth Revision, Clinical Modification (ICD-9-CM) codes, patients who underwent on-pump cardiac surgery were included from all patients in the MIMIC-III database. We only used the data from patients’ first ICU admission. Patients who missed ICU data were excluded. Patients with age < 18, and those who missed AG data at ICU admission or length of ICU stay < 24 h were also excluded.

### Data extraction

We extracted the data on the first day of ICU admission using Structured Query Language with pgAdmin4. The data included age, sex, body mass index (BMI), ethnicity, insurance, comorbidities [hypertension, diabetes, congestive heart failure, stroke, chronic obstructive pulmonary disease (COPD), kidney diseases, liver diseases, and malignancy], surgical type, sequential organ failure assessment (SOFA) score, simplified Acute Physiology Score II (SAPS II), laboratory data [leukocyte, platelet, hemoglobin, serum creatinine, serum urea, PH, base excess (BE), partial pressure of oxygen (PO_2_), partial pressure of carbon dioxide (PCO_2_), glucose, sodium, potassium, bicarbonate, and chloride], and vital signs [heart rate, respiratory rate, mean blood pressure (MBP), temperature, and SPO_2_]. The data of AG during the ICU stay were extracted. For serum AG, initial AG (Baseline AG) denoted the initial value of AG detected after surgery. The maximum and minimum AG denoted the maximum and minimum value of AG detected during postoperative ICU stay, respectively. For other laboratory parameters, the initial value of all measured values after surgery was used. All variables missed less than 20% of values in this study; multiple imputations were performed to impute missing values (13).

### Outcomes and follow-up

In-hospital mortality was the primary outcome. A total of 90-day and 4-year mortality, and length of stay (LOS) in ICU and hospital were considered as secondary outcomes. Follow-up duration was defined as the time from admission time (ADMITTIME) to the date of death (DOD_SNN) in MIMIC-III. We only set 4-year mortality as an outcome for 3,858 patients who were followed up for at least 4 years in the total cohort. The loss ratio of follow-up for 4-year mortality was 39.8% (2552/6410).

### Statistical analysis

All individuals classified into tertiles based on the initial AG levels detected after surgery (tertile 1: AG ≤ 11 mmol/L; tertile 2: 11 < AG ≤ 13 mmol/L; tertile 3: AG > 13 mmol/L). Continuous variables were expressed as median [interquartile range (IQR)] and the difference among groups was identified with non-parametric methods because of their skewed distribution. Categorical variables were expressed as number (percentage), and compared using the Chi-square or fisher test.

The generalized additive model (GAM) with smooth curve fitting was used to test the independent effects of the initial AG on in-hospital mortality with the fully adjusted model. Logistic regression models and Cox regression models were applied for estimating the association between postoperative AG (initial, maximum, and minimum) and mortality. Linear regression models were used to explore the relationship between the initial AG and LOS in ICU and hospital. Two different models were used in all regression analyses. Model 1 (the minimally adjusted model) was adjusted for age, sex, and ethnicity. Model 2 (the fully adjusted model) was adjusted for age, sex, ethnicity, BMI, insurance, hypertension, diabetes, congestive heart failure, stroke, COPD, kidney diseases, liver diseases, malignancy, surgical type, SOFA score, SAPS II score, leukocyte, platelet, hemoglobin, creatinine, urea, PH, BE, PO_2_, PCO_2_, glucose, heart rate, respiratory rate, MBP, temperature, and SPO_2_. Kaplan-Meier survival curves for 90-day and 4-year mortality were plotted and compared using the pairwise log-rank test. The receiver operating characteristic (ROC) curves depicted the predictive performance of postoperative AG for in-hospital, 90-day, and 4-year mortality.

Statistical analyses were conducted by the STATA (Version 14.0, StataCorp, College Station, TX, USA) and R 4.1.2 software.^1^ *P*-value < 0.05 was considered statistically significant.

### Sensitivity analysis

For sensitivity analyses, firstly, we conducted the subgroup analyses to ascertain the consistency of the relationship between the initial AG and in-hospital mortality in patients after cardiac surgery. Subgroup analyses were stratified by age strata (< 60 and ≥ 60), sex, BMI (< 25 and ≥ 25), hypertension, diabetes, congestive heart failure, COPD, and kidney diseases. Secondly, we also handled AG as a continuous variable in the multivariate regression analysis to assess the robustness of our study. Finally, we computed an *E*-value to evaluate the potential for unmeasured confounding between the initial AG and mortality (14). The *E*-value could quantify the required degree of an unmeasured confounding factor which could negate the discovered relationship between the initial AG and mortality.

To ensure the stability of the conclusion, we used different methods to group the cohort and reanalyzed. Because there are more patients in tertile 1 than in other tertiles, we divided tertile 1 into two groups according to the median of tertile 1 (10 mmol/L) for better comparability. Thus, the total cohort was divided into four groups (Group 1: AG ≤ 10 mmol/L; Group 2: AG = 11 mmol/L; Group 3: 11 < AG ≤ 13 mmol/L; Group 4: AG > 13 mmol/L). The relationship between AG groups and the primary outcome was examined using multivariable logistic regression with the same models. Additionally, we classified the total cohort according to AG quartiles (Quartile 1: AG ≤ 10 mmol/L; Quartile 2: 10 < AG ≤ 12 mmol/L; Quartile 3: 12 < AG ≤ 14 mmol/L; Quartile 4: AG > 14 mmol/L). The relationship between AG quartiles and the primary outcome was also explored using multivariable logistic regression with the same models.

## Results

### Population

**FIGURE 1:**
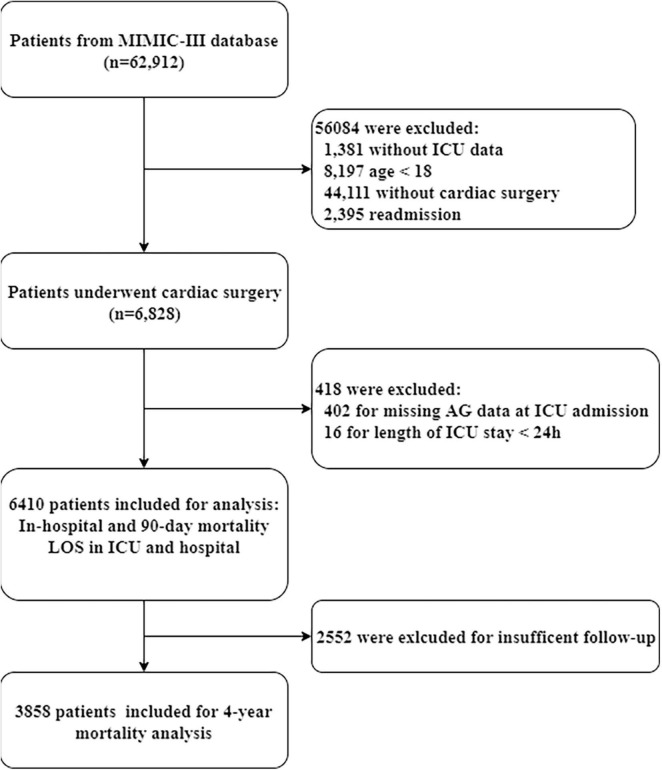
The flowchart of study cohort selection. LOS, length of stay.

During the study period, 6,828 adult patients who underwent cardiac surgery were identified in the MIMIC database. After excluding patients based on the exclusion criteria, the final cohort included 6,410 patients who met the selection criteria. The detailed information on study cohort selection is presented in Figure 1.

### Baseline characteristics

The baseline characteristics of the patients grouped by AG tertiles are summarized in Table 1. Among the 6,410 included patients, the median age (IQR) was 68.4 (59.6, 76.9), 1993 (31.1%) were female, and 4592 (71.6%) were white. Patients with higher AG were older, had higher BMI, and had more frequent medicare insurance. High AG patients also had higher proportions of diabetes, congestive heart failure, and kidney diseases, but had less frequent hypertension. Patients with higher AG had a higher SAPS II score on admission, while the SOFA score was similar among the groups. All patients were followed for at least 90 days; of these, 3,858 were followed for at least 4 years.

**TABLE 1:** Baseline characteristics according to the tertile of the initial AG.

	Total cohort	Tertile 1 (AG ≤ 11)	Tertile 2 (11 < AG ≤ 13)	Tertile 3 (AG > 13)	*P*-value
*n*	6410	3061	1701	1648	
**Demographics**					
Age (years)	68.4 (59.6, 76.9)	67.8 (59.3, 76.5)	68.7 (59.6, 77.1)	69.7 (59.9, 77.2)	0.006
Female, *n* (%)	1993 (31.1%)	939 (30.7%)	519 (30.5%)	535 (32.5%)	0.375
BMI	27.9 (24.6, 31.8)	27.4 (24.5, 31.2)	28.1 (24.9, 32.1)	28.4 (24.8, 32.4)	< 0.001
Ethnicity, *n* (%)					< 0.001
White	4592 (71.6)	2262 (73.9)	1209 (71.1)	1121 (68)	
Black	183 (2.9)	79 (2.6)	42 (2.5)	62 (3.8)	
Asian	115 (1.8)	62 (2)	27 (1.6)	26 (1.6)	
Other	1520 (23.7)	658 (21.5)	423 (24.9)	439 (26.6)	
Insurance, *n* (%)					0.001
Medicare	3689 (57.6)	1691 (55.2)	981 (57.7)	1017 (61.7)	
Private	2290 (35.7)	1153 (37.7)	605 (35.6)	532 (32.3)	
Others	431 (6.7)	217 (7.1)	115 (6.8)	99 (6)	
**Comorbidities, *n* (%)**					
Hypertension	3931 (61.3)	1995 (65.2)	1092 (64.2)	844 (51.2)	< 0.001
Diabetes	2145 (33.5)	950 (31)	564 (33.2)	631 (38.3)	< 0.001
Congestive heart failure	1928 (30.1)	762 (24.9)	499 (29.3)	667 (40.5)	< 0.001
Stroke	129 (2.0)	60 (2)	29 (1.7)	40 (2.4)	0.317
COPD	1302 (20.3)	660 (21.6)	327 (19.2)	315 (19.1)	0.059
Kidney diseases	595 (9.3)	194 (6.3)	152 (8.9)	249 (15.1)	< 0.001
Liver diseases	73 (1.1)	30 (1)	23 (1.4)	20 (1.2)	0.483
Malignancy	184 (2.9)	84 (2.7)	47 (2.8)	53 (3.2)	0.622
Surgical type, *n* (%)					0.063
Valvular surgery	1711 (26.7)	839 (27.4)	477 (28)	395 (24)	
CABG	3745 (58.4)	1765 (57.7)	982 (57.7)	998 (60.6)	
CABG + valvular surgery	954 (14.9)	457 (14.9)	242 (14.2)	255 (15.5)	
**Severity scale**					
SOFA	4.0 (3.0, 6.0)	4.0 (3.0, 6.0)	4.0 (3.0, 6.0)	4.0 (3.0, 7.0)	0.399
SAPS II	34.0 (27.0, 41.0)	33.0 (27.0, 40.0)	34.0 (27.0, 41.0)	35.0 (28.0, 44.0)	< 0.001
**Laboratory events**					
Leukocyte, 10^9^/L	12.0 (9.1, 15.5)	11.9 (9.3, 15.4)	12.0 (9.0, 15.4)	12.1 (9.0, 15.9)	0.592
Platelet, 10^9^/L	162.0 (126.0, 209.0)	152.0 (123.0, 191.0)	167.0 (126.0, 214.0)	183.0 (136.0, 244.0)	< 0.001
Hemoglobin, g/dl	9.9 (8.5, 11.4)	9.7 (8.3, 11.0)	9.9 (8.6, 11.3)	10.5 (8.9, 12.1)	< 0.001
Serum creatinine, mg/dl	0.9 (0.7, 1.1)	0.8 (0.7, 1.0)	0.9 (0.7, 1.1)	1.0 (0.8, 1.5)	< 0.001
Serum urea, mg/dl	16.0 (13.0, 22.0)	15.0 (12.0, 19.0)	16.0 (13.0, 21.0)	20.0 (14.0, 31.0)	< 0.001
PH	7.4 (7.4, 7.4)	7.4 (7.4, 7.5)	7.4 (7.3, 7.5)	7.4 (7.3, 7.4)	0.005
BE	0.0 (−1.0, 2.0)	1.0 (0.0, 2.2)	0.0 (−1.0, 2.0)	0.0 (−1.7, 2.0)	< 0.001
PO_2_, mmHg	322.0 (237.0, 389.0)	337.0 (267.0, 396.0)	321.0 (238.0, 390.0)	286.2 (175.3, 367.0)	< 0.001
PCO_2_, mmHg	40.4 (36.0, 45.0)	41.0 (37.0, 45.0)	41.0 (36.0, 45.0)	40.0 (36.0, 44.0)	< 0.001
Glucose, mg/dl	135.0 (112.0, 162.0)	134.0 (113.0, 158.0)	134.0 (112.0, 160.0)	139.0 (113.0, 173.0)	< 0.001
Sodium, mmol/L	137.0 (135.0, 139.0)	136.0 (135.0, 138.0)	137.0 (135.0, 139.0)	137.0 (135.0, 140.0)	< 0.001
Potassium, mmol/L	4.3 (3.9, 4.9)	4.4 (3.9, 5.0)	4.3 (3.8, 4.9)	4.2 (3.8, 4.8)	< 0.001
Bicarbonate, mmol/L	24.0 (22.0, 26.0)	24.0 (23.0, 26.0)	24.0 (22.0, 25.0)	23.0 (21.0, 25.0)	< 0.001
Chloride, mmol/L	107.0 (104.0, 110.0)	108.0 (106.0, 111.0)	107.0 (105.0, 110.0)	106.0 (102.0, 109.0)	< 0.001
**Vital signs**					
HR, beats/min	83.0 (76.0, 90.0)	82.0 (77.0, 88.0)	83.0 (75.0, 90.0)	84.0 (76.0, 92.0)	< 0.001
RR, times/min	14.0 (12.0, 16.0)	14.0 (12.0, 16.0)	14.0 (12.0, 16.0)	15.0 (12.0, 19.8)	< 0.001
MBP, mmHg	70.0 (63.0, 79.0)	69.0 (62.0, 76.7)	71.0 (63.3, 80.0)	73.0 (64.3, 83.0)	< 0.001
Temperature, °C	36.1 (35.6, 36.6)	36.1 (35.6, 36.5)	36.1 (35.6, 36.7)	36.2 (35.7, 36.8)	< 0.001
SPO_2_, %	100.0 (99.0, 100.0)	100.0 (100.0, 100.0)	100.0 (99.0, 100.0)	100.0 (97.0, 100.0)	< 0.001

All data were shown as median and interquartile range, or number and percentage.

BMI, body mass index; COPD, chronic obstructive pulmonary disease; CABG, coronary artery bypass grafting; SOFA, Sequential Organ Failure Assessment; SAPS II, Simplified Acute Physiology Score; BE, base excess; PO_2_, partial pressure of oxygen; PCO_2_, partial pressure of carbon dioxide; HR, heart rate; RR, respiratory rate; MBP, mean blood pressure.

The outcomes across tertiles of initial AG are provided in Table 2. The in-hospital mortality was 2.2% (143/6410); as AG tertiles increased, the in-hospital mortality increased gradually. For secondary outcomes, the 90-day mortality and 4-year mortality were 4.7% (299/6410) and 17.9% (691/3858), respectively; as AG tertiles increased, both 90-day mortality and 4-year mortality increased stepwise from tertile 1 to tertile 3. The LOS in ICU and hospital also increased as AG tertiles increased.

**TABLE 2:** Outcomes of patients across the tertile of the initial AG.

	Total cohort	Tertile 1 (AG ≤ 11)	Tertile 2 (11 < AG ≤ 13)	Tertile 3 (AG > 13)	*P*-value
*n*	6410	3061	1701	1648	
In-hospital mortality	143 (2.2)	19 (0.6)	26 (1.5)	98 (5.9)	< 0.001
90-days mortality	299 (4.7)	64 (2.1)	64 (3.8)	171 (10.4)	< 0.001
ICU LOS (days)	2.2 (1.3, 4.0)	2.1 (1.2, 3.2)	2.3 (1.3, 4.1)	3.1 (1.8, 5.8)	< 0.001
Hospital LOS (days)	7.7 (5.3, 11.2)	7.0 (5.2, 9.9)	7.8 (5.4, 11.2)	9.1 (6.1, 14.2)	< 0.001
*n*	3858	1502	1154	1202	
4-year mortality^a^	691 (17.9)	176 (11.7)	195 (16.9)	320 (26.6)	< 0.001

All data were shown as median and interquartile range, or number and percentage.

^a^4-year mortality was set as an outcome for 3,858 patients who were followed up for at least 4 years in the total cohort. LOS, length of stay.

### Postoperative anion gap and the risk of in-hospital mortality

**FIGURE 2:**
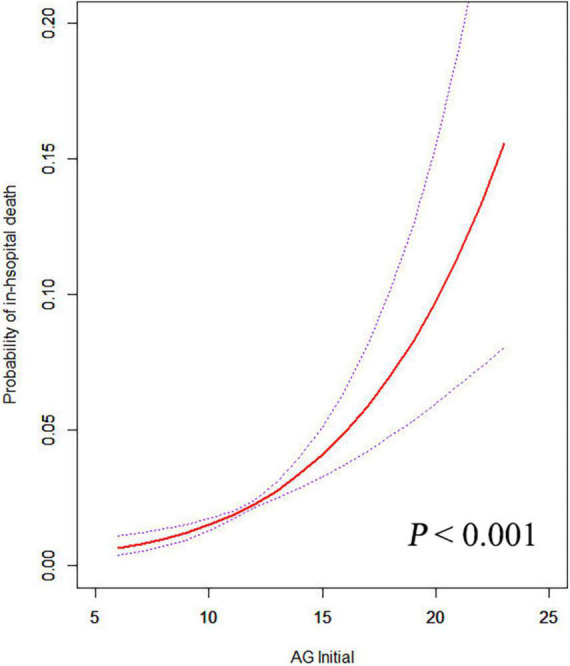
The relationship between the initial AG and in-hospital mortality. A positive relationship between AG and the risk of in-hospital mortality was observed by the general additive model after adjusting for age, sex, ethnicity, BMI, insurance, hypertension, diabetes, congestive heart failure, stroke, COPD, kidney disease, liver disease, malignancy, type of surgery, SOFA score, SAPS II score, leukocyte, platelet, hemoglobin, creatinine, urea, PH, base excess, PO_2_, PCO_2_, glucose, heart rate, respiratory rate, MBP, temperature, and SPO_2_.

The relationship between initial AG levels and the risk of in-hospital mortality was shown by the smooth curve fitting (Figure 2). Taking the initial AG as a continuous variable, we observed a significant positive relationship between AG and in-hospital mortality (*P* < 0.001), even after adjusting for all potential covariates.

We further examined this finding using multivariate logistic regression analyses with defined models (with the tertile 1 as reference) (Table 3). Even after adjusting for demographics, comorbidities, severity scale, and biochemical examination (Model 2, the fully adjusted model), high tertile of AG was an independent risk factor for in-hospital mortality. The adjusted OR for middle (tertile 2) and high (tertile 3) AG group were 2.05 (95% CI 1.11–3.76, *P* = 0.021) and 4.51 (95% CI 2.57–7.91, *P* < 0.001), compared with the tertile 1 group. *P* for trend < 0.001. When taking initial AG as a continuous variable, the association remained significant in the fully adjusted model (OR 1.23, 95% CI 1.15–1.31, *P* < 0.001).

**TABLE 3:** The association between postoperative AG and outcomes.

	unadjusted		Model 1		Model 2	
	OR (95% CI)	*P*-value	OR (95% CI)	*P*-value	OR (95% CI)	*P*-value
**In-hospital mortality**						
**Initial AG**						
Tertile 1	1.00		1.00		1.00	
Tertile 2	2.49 (1.37–4.50)	0.003	2.43 (1.34–4.40)	0.003	2.05 (1.11–3.76)	0.021
Tertile 3	10.12 (6.17–16.61)	< 0.001	9.62 (5.85–15.80)	< 0.001	4.51 (2.57–7.91)	< 0.001
*P* for trend		< 0.001		< 0.001		< 0.001
Initial AG (mmol/L)	1.38 (1.31–1.44)	< 0.001	1.37 (1.31–1.44)	< 0.001	1.23 (1.15–1.31)	< 0.001
Max AG (mmol/L)	1.46 (1.41–1.52)	< 0.001	1.47 (1.42–1.53)	< 0.001	1.46 (1.39–1.53)	< 0.001
Min AG (mmol/L)	1.31 (1.23–1.40)	< 0.001	1.31 (1.22–1.39)	< 0.001	1.15 (1.07–1.25)	< 0.001

	**HR (95% CI)**	***P*-value**	**HR (95% CI)**	***P*-value**	**HR (95% CI)**	***P*-value**

**90-day mortality**						
**Initial AG**						
Tertile 1	1.00		1.00		1.00	
Tertile 2	1.82 (1.28,2.57)	< 0.001	1.79 (1.26–2.53)	0.001	1.48 (1.04–2.10)	0.029
Tertile 3	5.21 (3.91,6.95)	< 0.001	4.98 (3.73–6.64)	< 0.001	2.51 (1.80–3.50)	< 0.001
*P* for trend		< 0.001		< 0.001		< 0.001
Initial AG (mmol/L)	1.27 (1.23,1.31)	< 0.001	1.26 (1.22–1.3)	< 0.001	1.13 (1.09–1.18)	< 0.001
**4-year mortality**						
**Initial AG**						
Tertile 1	1.00		1.00		1.00	
Tertile 2	1.50 (1.22–1.84)	< 0.001	1.46 (1.19–1.79)	< 0.001	1.25 (1.02–1.54)	0.033
Tertile 3	2.55 (2.12–3.07)	< 0.001	2.42 (2.01–2.91)	< 0.001	1.48 (1.20–1.82)	< 0.001
*P* for trend		< 0.001		< 0.001		< 0.001
Initial AG (mmol/L)	1.20 (1.17–1.23)	< 0.001	1.19 (1.16–1.22)	< 0.001	1.09 (1.06–1.12)	< 0.001

	**β (95% CI)**	***P*-value**	**β (95% CI)**	***P*-value**	**β (95% CI)**	***P*-value**

**ICU LOS**						
**Initial AG**						
Tertile 1	0.00		0.00		0.00	
Tertile 2	1.03 (0.70–1.36)	< 0.001	1.00 (0.67–1.33)	< 0.001	0.82 (0.50–1.14)	< 0.001
Tertile 3	2.74 (2.41–3.08)	< 0.001	2.68 (2.35–3.01)	< 0.001	1.80 (1.44–2.15)	< 0.001
*P* for trend		< 0.001		< 0.001		< 0.001
Initial AG (mmol/L)	0.44 (0.40–0.49)	< 0.001	0.43 (0.39–0.48)	< 0.001	0.31 (0.25–0.36)	< 0.001
**Hospital LOS**						
**Initial AG**						
Tertile 1	0.00		0.00		0.00	
Tertile 2	1.47 (1.02–1.92)	< 0.001	1.43 (0.98–1.88)	< 0.001	1.12 (0.69–1.54)	< 0.001
Tertile 3	3.3 (2.85–3.75)	< 0.001	3.21 (2.75–3.66)	< 0.001	1.74 (1.26–2.22)	< 0.001
*P* for trend		< 0.001		< 0.001		< 0.001
Initial AG (mmol/L)	0.53 (0.47–0.6)	< 0.001	0.52 (0.46–0.58)	< 0.001	0.28 (0.21–0.35)	< 0.001

The tertile 1 group was set as the reference group when AG served as a categorical variable in regression models.

Adjusted covariates:

Model 1 = age, sex, ethnicity.

Model 2 = Model 1 plus (BMI, insurance, hypertension, diabetes, congestive heart failure, stroke, COPD, kidney disease, liver disease, malignancy, type of surgery, SOFA score, SAPS II score, leukocyte, platelet, hemoglobin, creatinine, urea, PH, BE, PO_2_, PCO_2_, glucose, heart rate, respiratory rate, MBP, temperature, Spo_2_). AG, anion gap; LOS, length of stay; BMI, body mass index; COPD, chronic obstructive pulmonary disease; SOFA, Sequential Organ Failure Assessment; SAPS II, Simplified Acute Physiology Score; PO_2_, partial pressure of oxygen; PCO_2_, partial pressure of carbon dioxide; MBP, mean blood pressure.

We also assessed the associations between maximum and minimum AG during postoperative ICU stay and in-hospital mortality. Taking maximum and minimum AG as continuous variables, we found that either maximum AG or minimum AG was associated with in-hospital mortality independent of all potential covariates. The adjusted OR for maximum AG and minimum AG were 1.46 (95% CI 1.39–1.53, *P* < 0.001) and 1.15 (95% CI 1.07–1.25, *P* < 0.001) in the fully adjusted model, respectively.

### Relationship between initial anion gap and secondary outcomes

**FIGURE 3:**
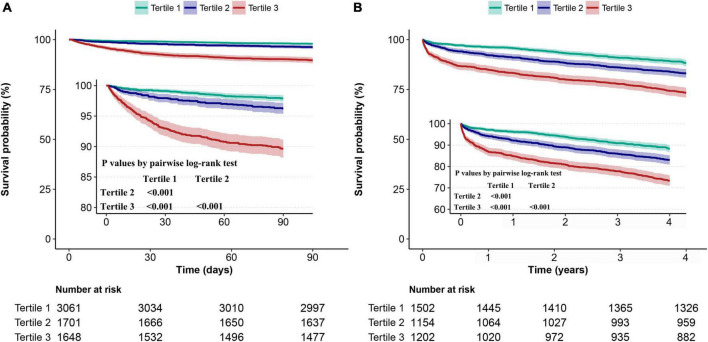
Kaplan-Meier survival analysis among patients stratified by the tertile of the initial AG. The comparisons of **(A)** 90-day and **(B)** 4-year survival of the three tertiles were made by the pairwise log-rank test.

Kaplan-Meier survival curves stratified by AG tertiles reveal a positive association between initial AG levels and 90-day and 4-year mortality (Figure 3). Patients with higher AG had lower 90-day and 4-year survival rates (all *P* < 0.001, pairwise log-rank test).

Moreover, we performed multivariate Cox proportional hazard analyses to evaluate the independent prognostic value of initial AG on 90-day and 4-year mortality. The proportional hazards assumption was also evaluated and verified. In the minimally and fully adjusted model, the risk of 90-day and 4-year mortality enhanced as AG tertiles increased. In the fully adjusted model, the adjusted HR of 90-day mortality for participants in AG tertile 2–3 were 1.48 (95% CI 1.04–2.10, *P* = 0.029) and 2.51 (95% CI 1.80–3.50, *P* < 0.001), respectively, compared with those in tertile 1 (*P* for trend < 0.001). The adjusted HR of 4-year mortality for participants in AG tertile 2–3 were 1.25 (95% CI 1.02–1.54, *P* = 0.033) and 1.48 (95% CI 1.20–1.82, *P* < 0.001), respectively, compared with those in tertile 1 (*P* for trend < 0.001). The correlation between initial AG and 90-day and 4-year mortality remained significant when initial AG entered the fully adjusted model as a continuous variable.

We also used multivariate linear regression analyses to assess the relationship between initial AG and LOS in ICU and hospital. Similarly, higher AG levels were associated with longer LOS even after adjusting for all potential covariates (Table 3).

### Predictive ability of postoperative anion gap for mortality

**FIGURE 4:**
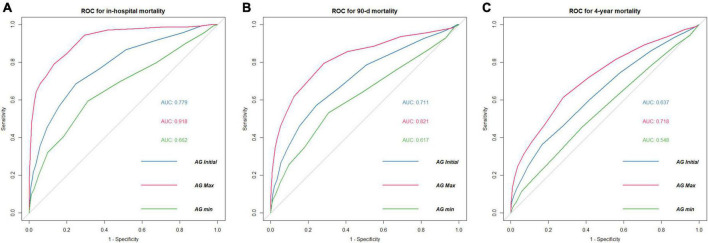
ROC curves of postoperative serum AG for mortality. **(A)** In-hospital mortality **(B)** 90-day mortality **(C)** 4-year mortality. AUC, area under curve; ROC, receiver operating characteristic; AG Initial, the initial value of AG after surgery; AG Max, the maximum value of AG during postoperative ICU stay; AG Min, the minimum value of AG during postoperative ICU stay.

The predictive performance of postoperative AG for in-hospital, 90-day and 4-year mortality was assessed using ROC curves (Figure 4). The initial AG showed moderately good predictive performance for in-hospital, 90-day, and 4-year mortality. Notably, the maximum AG during ICU stay demonstrated an excellent predictive value for in-hospital mortality with an AUC of 0.918. However, the AUC of AG showed a decreasing trend with increasing follow-up.

### Sensitivity analysis

**FIGURE 5:**
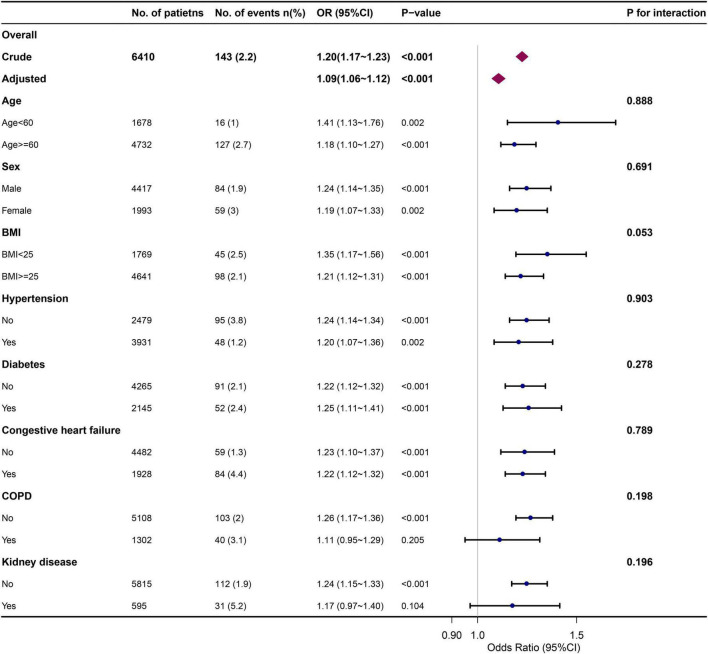
Association between initial AG and in-hospital mortality in different subgroups. Each stratification analysis adjusted for all the confounding factors (age, sex, ethnicity, BMI, insurance, hypertension, diabetes, congestive heart failure, stroke, COPD, kidney disease, liver disease, malignancy, type of surgery, SOFA score, SAPS II score, leukocyte, platelet, hemoglobin, creatinine, urea, PH, base excess, PO_2_, PCO_2_, glucose, heart rate, respiratory rate, MBP, temperature, SPO_2_) except the stratification factor itself.

The subgroup analysis was conducted to ascertain the consistency of the relationship between the initial AG and in-hospital mortality (Figure 5). Though subgroup analysis was performed according to confounders including age, sex, BMI, hypertension, diabetes, congestive heart failure, COPD, and kidney diseases, no significant interaction was observed in the subgroup (*P* for interaction > 0.05 for all).

As shown above, apart from studying the association between AG tertiles and mortality, we also used continuous variable of AG for multivariable analysis and yielded similar findings. In addition, we computed an *E*-value to evaluate the sensitivity to unmeasured confounders. We found that the observed OR of 4.51 (tertile 3 vs. tertile 1, Table 3) for in-hospital mortality could be explained away by an unmeasured confounder with an OR of at least 8.49, which suggested that unmeasured confounding was difficult to negate the observed association.

To ensure the stability of the conclusion, we used different methods to group the cohort and reanalyzed. Because there are more patients in tertile 1 than in other tertiles, we divided tertile 1 into two groups according to the median of tertile 1 (10 mmol/L) for better comparability. Thus, the total cohort was divided into four groups (Group 1: AG ≤ 10 mmol/L; Group 2: AG = 11 mmol/L; Group 3: 11 < AG ≤ 13 mmol/L; Group 4: AG > 13 mmol/L). The relationship between AG groups and in-hospital mortality was examined using multivariable logistic regression with the same models (Supplementary Table 1). Group 2 had an insignificant influence on in-hospital mortality compared with group 1; while group 3 and group 4 showed an elevated risk of in-hospital mortality.

In addition, we classified the total cohort into four groups according to AG quartiles (Quartile 1: AG ≤ 10 mmol/L; Quartile 2: 10 < AG ≤ 12 mmol/L; Quartile 3: 12 < AG ≤ 14 mmol/L; Quartile 4: AG > 14 mmol/L). The relationship between AG quartiles and in-hospital mortality was also explored using multivariable logistic regression with the same models (Supplementary Table 2). Patients in quartile 3 and 4 had an enhanced risk of in-hospital mortality compared with those in quartile 1, which was similar to the results analyzed using the AG tertiles grouping.

## Discussion

In this retrospective cohort study, high postoperative AG was associated with an increased risk of in-hospital mortality after cardiac surgery. This correlation was similarly observed for the secondary outcomes of postoperative 90-day and 4-year mortality. We also found a positive association between postoperative AG and LOS in ICU and hospital. Additionally, the AG levels – including initial and maximum AG values during ICU stay – had a high diagnostic performance for in-hospital, 90-day, and 4-year mortality after cardiac surgery.

Acid-base balance is essential for maintaining metabolic homeostasis and physiological function. As a traditional index of acid-base balance, AG may be a prognostic maker to predict outcomes. Previous studies demonstrated that AG was related to mortality of many cardiovascular diseases (6, 9, 15) and patients with elevated AG showed worse cardiac (16) and renal functions (17). These evidences suggested that AG might be an indicator of severe disease states. However, the role of AG in patients after cardiac surgery has been little discussed. Our study extends these findings by revealing that postoperative elevated AG was associated with an increase in short- and long-term mortality in patients after cardiac surgery.

Regrettably, the mechanism of how AG levels affect patients’ prognosis remains unclear. One possible explanation is that increased AG indicates a status of hypoperfusion in the body. Although the correlation between inadequate perfusion and increased AG levels has been elucidated in different settings, how AG plays a role in indicating a state of hypoperfusion following cardiac surgery is uncertain. Postoperative acid-base imbalance and electrolyte disturbances are common in patients who underwent cardiac surgery (18). Under cardiopulmonary bypass, hypoxia, ischemia, and acidosis occur frequently, which promote increased acidic substance and thus elevate AG levels (15). AG may indicate the status of hypoperfusion by reflecting the cumulative levels of acidic substance due to ischemia; while hemodynamic instability like hypotension is demonstrated to be associated with prognosis. With the aggravation of ischemia, AG levels are further increased and forecast a poor outcome. In this study, postoperative AG was related to short- and long-term mortality after adjustment of potential confounders. We also found that patients in the high AG group had relatively high SAPS II scores, which indicated a possible correlation between high AG and the severe disease condition. Although the detailed mechanism of how increased AG indicates severe disease status needs further study, AG may be associated with the postoperative ischemic conditions and predicts mortality following cardiac surgery.

Another explanation is that elevated AG may predict organ dysfunction. Previous studies reported that AG level was related to cardiac function (16). In the coronary heart disease cohort, patients with high AG had worse cardiac function and higher mortality. Notably, cardiac dysfunction is common and contributes to postoperative morbidity and mortality after on-pump cardiac surgery, which is also called “Low cardiac output syndrome.” The etiology of postoperative cardiac dysfunction is complex and is reported to be associated with myocardial ischemia, operative injury, reperfusion injury, and preexisting cardiac disease (19). Studies showed that in the heart failure population, mortality was more frequent in patients with high AG levels (9). In this study, we also found that patients in the higher AG group presented more often history of congestive heart failure. Postoperative high AG level may be a sign of cardiac dysfunction and predicts a worse prognosis.

Additionally, we found that postoperative renal function was negatively correlated with AG level, as patients in the high AG group had a relatively high level of serum creatinine and urea (Table 1). Studies reported that 20–30% of patients undergoing cardiac surgery developed acute kidney injury (AKI), and it was related to increased risk of morbidity and mortality, both in the short- and long-term (20). In the AKI cohort, a U-shaped curve relationship was observed between serum AG level and 30-day mortality, while higher AG was a significant predictor of short- and long-term mortality independent of various confounders (21). A study revealed that patients suffering from chronic kidney disease had greater AG, and AG was associated with the progression to end-stage renal disease (22). Patients with high AG level following cardiac surgery was likely to have a worse renal function and even develop AKI. Since AKI is a traditional risk factor for mortality in the critically ill population, patients with greater AG levels and decreased renal function had an elevated risk of mortality following cardiac surgery.

Few studies have investigated the utility of AG for predicting mortality in patients who underwent cardiac surgery. Based on the analysis of a large-scale sample, this research suggested that serum AG level could be a prognostic indicator for patients following cardiac surgery. Despite there is a normal range of serum AG (8–16 mmol/L), the clinical cutoff value may vary when taking AG as a prognostic index in different populations. In our study, we observed a positive relationship between postoperative initial AG level and in-hospital mortality (Figure 2). Compared with AG ≤ 11 mmol/L, AG > 11 mmol/L was an independent risk factor for in-hospital, 90-day, and 4-year mortality after multivariate adjustment (Table 3). Both the initial AG level after cardiac surgery and the maximum AG level during ICU stay presented a satisfactory diagnostic performance for in-hospital and 90-day mortality. Thus, for patients with AG level > 11 mmol/L after cardiac surgery, we may need to actively search the etiology of high AG and provide corresponding therapy.

This research has important limitations. First, despite the sample size is large, this is a single-center retrospective research. Potential bias and confounders may affect the results although we used different models to confirm the independent effect of AG on mortality. Therefore, we further conduct the *E*-value sensitivity analysis to quantify the potential degree of unmeasured confounders and found that an unmeasured confounder was improbable to negate the observed association between AG and in-hospital mortality. Second, we only included the initial, maximum, and minimum AG value following cardiac surgery for analysis. Whether repeated measurements of AG have additional information for prognosis is unclear. This should be valued in the next step of our research. Third, we did not include lactate and ketone body for analysis because the missing data exceeded 20%. We did not know whether these indexes showed better diagnostic power than AG, and the relationship between these indexes and AG in patients undergoing cardiac surgery was also unclear. Forth, some valuable information was also lacking in the database, like intraoperative details. These were the deficiencies of the MIMIC-III itself. Fifth, although AG showed satisfactory diagnostic ability, the AG alone was insufficient to predict prognosis. For long-term survival, the AUC of AG decreased. This was reasonable as increasing death events caused by other unknown confounders occurred over time, and if patients could survive the primal internal environment disturbance, they were probable to have more long-term survival. However, as we observed a significant correlation between elevated AG and poor outcomes, we suggested that clinicians should notice a higher risk of death in this situation and researchers should appreciate AG when constructing new prediction models in patients following cardiac surgery. The causal association between postoperative AG and mortality warrants a prospective investigation.

In conclusion, this cohort study suggested that postoperative elevated AG levels may be associated with increased short- and long-term mortality among patients who underwent cardiac surgery.

## Data availability statement

The raw data supporting the conclusions of this article will be made available by the authors, without undue reservation.

## Ethics statement

MIMIC-III database used in the present study was approved by the Institutional Review Boards (IRB) of the Massachusetts Institute of Technology and does not contain protected health information. Written informed consent for participation was not required for this study in accordance with the national legislation and the institutional requirements.

## Author contributions

YL and JL conducted the statistical analysis. YL drafted the manuscript. LW and JL revised the manuscript. YT drew the figures. YT, JC, and XC collected the data. YL and HH designed the study and reviewed the manuscript. All authors approved the final version of the manuscript.

## Footnotes

1
http://www.R-project.org
