# Supplementary material for: Postoperative anion gap associates with short- and long-term mortality after cardiac surgery: A large-scale cohort study
Source: Front Cardiovasc Med. 2022 Oct 12;9:1024484. doi: 10.3389/fcvm.2022.1024484 (PMC9596785; doi:10.3389/fcvm.2022.1024484)
Supplement: Supplementary file 1 [file Table_1.DOCX]

**Table S1. The association between postoperative AG groups and primary outcomes.**

|  | **unadjusted** | |  | **Model 1** | |  | **Model 2** | |
| --- | --- | --- | --- | --- | --- | --- | --- | --- |
|  | **OR (95%CI)** | ***P*-value** |  | **OR (95%CI)** | ***P*-value** |  | **OR (95%CI)** | ***P*-value** |
| **In-hospital mortality** |  |  |  |  |  |  |  |  |
| Initial AG |  |  |  |  |  |  |  |  |
| Group 1 | 1.00 |  |  | 1.00 |  |  | 1.00 |  |
| Group 2 | 1.19(0.47~3.04) | 0.709 |  | 1.16(0.45~2.95) | 0.758 |  | 1.02(0.39~2.62) | 0.973 |
| Group 3 | 2.64(1.33~5.26) | 0.006 |  | 2.56(1.29~5.09) | 0.007 |  | 2.07(1.03~4.18) | 0.042 |
| Group 4 | 10.77(5.89~19.68) | <0.001 |  | 10.13(5.53~18.54) | <0.001 |  | 5.39(2.82~10.31) | <0.001 |
| *P* for trend |  | <0.001 |  |  | <0.001 |  |  | <0.001 |

The Group 1 was set as the reference group when AG served as a categorical variable in regression models.

Adjusted covariates:

Model 1 = age, sex, ethnicity

Model 2 = Model 1 plus (BMI, insurance, hypertension, diabetes, congestive heart failure, stroke, COPD, kidney disease, liver disease, malignancy, type of surgery, SOFA score, SAPS II score, leukocyte, platelet, hemoglobin, creatinine, urea, PH, BE, PO2, PCO2, glucose, heart rate, respiratory rate, MBP, temperature, Spo2)

AG, anion gap; BMI, body mass index; COPD, chronic obstructive pulmonary disease; SOFA, Sequential Organ Failure Assessment; SAPS II, Simplified Acute Physiology Score; PO2, partial pressure of oxygen; PCO2, partial pressure of carbon dioxide; MBP, mean blood pressure

**Table S2. The association between postoperative AG quartiles and primary outcomes.**

|  | **unadjusted** | |  | **Model 1** | |  | **Model 2** | |
| --- | --- | --- | --- | --- | --- | --- | --- | --- |
|  | **OR (95%CI)** | ***P*-value** |  | **OR (95%CI)** | ***P*-value** |  | **OR (95%CI)** | ***P*-value** |
| **In-hospital mortality** |  |  |  |  |  |  |  |  |
| Initial AG |  |  |  |  |  |  |  |  |
| Quartile 1 | 1.00 |  |  | 1.00 |  |  | 1.00 |  |
| Quartile 2 | 1.96 (0.97~3.98) | 0.061 |  | 1.91 (0.94~3.87) | 0.073 |  | 1.69 (0.83~3.44) | 0.152 |
| Quartile 3 | 3.61 (1.83~7.12) | <0.001 |  | 3.43 (1.74~6.78) | <0.001 |  | 2.57 (1.28~5.19) | 0.008 |
| Quartile 4 | 13.91 (7.55~25.62) | <0.001 |  | 13.02 (7.06~24.02) | <0.001 |  | 5.17 (2.6~10.29) | <0.001 |
| *P* for trend |  | <0.001 |  |  | <0.001 |  |  | <0.001 |

The quartile 1 group was set as the reference group when AG served as a categorical variable in regression models.

Adjusted covariates:

Model 1 = age, sex, ethnicity

Model 2 = Model 1 plus (BMI, insurance, hypertension, diabetes, congestive heart failure, stroke, COPD, kidney disease, liver disease, malignancy, type of surgery, SOFA score, SAPS II score, leukocyte, platelet, hemoglobin, creatinine, urea, PH, BE, PO2, PCO2, glucose, heart rate, respiratory rate, MBP, temperature, Spo2)

AG, anion gap; BMI, body mass index; COPD, chronic obstructive pulmonary disease; SOFA, Sequential Organ Failure Assessment; SAPS II, Simplified Acute Physiology Score; PO2, partial pressure of oxygen; PCO2, partial pressure of carbon dioxide; MBP, mean blood pressure
